# Structural, optical and biological characterization of a new cobalt-based mixed halide hybrid compound: insights from DFT and vibrational analysis

**DOI:** 10.1098/rsos.250485

**Published:** 2025-07-23

**Authors:** Iteb Ben Mahmoud, Naoufel Ben Hamadi, Sandra Walha, Nourhene Zammel, Ali Ben Ahmed, Ahlem Guesmi, Wesam Abd El-Fattah, Ferdinando Costantino, Houcine Naïli

**Affiliations:** ^1^ Laboratory Physical-Chemistry of the Solid State, Department of Chemistry, Faculty of Sciences of Sfax, University of Sfax, BP 1171, Sfax 3000, Tunisia; ^2^ Chemistry Department, College of Science, Imam Mohammad Ibn Saud Islamic University (IMSIU), P.O. Box 5701, Riyadh 11432, Saudi Arabia; ^3^ University of Sfax, Faculty of Medicine of Sfax, Laboratory of Histo-Embryology and Cytogenetics, Sfax, Tunisia; ^4^ University of Sfax, Faculty of Sciences of Sfax, Department of Physics, Laboratory of Applied Physics, Sfax, Tunisia; ^5^ Department of Chemistry Biology and Biotechnologies, University of Perugia Via Elce di Sotto 8, 06123 Perugia, Italy

**Keywords:** cobalt-based hybrid material, density functional theory, anti-bacterial, alpha-amylase activity, anti-inflammatory effects

## Abstract

A novel cobalt-based hybrid compound, bis(4-dimethylaminopyridinium)bromo-chlorocobaltate(II) (C_7_H_11_N_2_)_2_[CoBr_2.28_Cl_1.72_] (1), was successfully synthesized via a slow evaporation method at room temperature. The crystalline material was thoroughly characterized using single-crystal X-ray diffraction, Hirshfeld surface analysis, Fourier transform infrared (FT-IR) and Raman spectroscopy, UV–Vis optical absorption, thermal analysis and theoretical density functional theory (DFT) calculations. Crystallographic analysis revealed that compound (1) crystallizes in the centrosymmetric monoclinic space group *C*2/*c*, featuring discrete [CoBr_2.28_Cl_1.72_]^2-^ anions and protonated amine cations (C₇H₁₁N₂)^+^. These units are interconnected via an extensive network of N–H···Br/Cl and C–H···Br/Cl hydrogen bonds, stabilizing the crystal lattice. Thermal analysis indicated a significant phase transition at approximately 203°C, underscoring the thermal responsiveness of the structure. Spectroscopic techniques highlighted the vibrational characteristics of key functional groups, and DFT calculations offered further insights into electronic and structural properties. Biological evaluation demonstrated that compound (1) possesses notable antimicrobial activity, particularly against *Escherichia coli*, *Staphylococcus aureus* and *Bacillus subtilis*, with inhibition zones ranging from moderate to strong depending on the microorganism tested. Additionally, the compound exhibited promising antioxidant capacity in 1,1-diphenyl-2-picrylhydrazyl (DPPH) assays, moderate α-amylase inhibition indicative of anti-diabetic potential and measurable anti-inflammatory effects. These findings suggest that compound (1) is a multifunctional material with potential applications in biomedical and pharmaceutical fields.

## 1. Introduction

Over the past few decades, organic–inorganic hybrid materials have emerged as a fascinating class of compounds, capturing the sustained interest of researchers across various disciplines. Their appeal lies in the seamless integration of the rigid, highly ordered architecture of inorganic frameworks with the flexible, tunable functionalities of organic moieties [1–3]. This combination not only enhances structural and thermal stability but also opens avenues for precise control over physico-chemical properties. The interplay between organic and inorganic components fosters a rich network of weak non-covalent interactions such as hydrogen bonding, π–π stacking, van der Waals forces and electrostatic interactions, which play a crucial role in dictating the material’s overall behaviour. As a result, these hybrids exhibit a wide spectrum of technologically relevant properties, including but not limited to magnetic ordering, efficient luminescence, second-order nonlinear optical (NLO) responses, enhanced electrical conductivity and switchable ferroelectric behaviour [3–12]. A growing class within this field involves hybrid salts of the general formula A_2_MX_4_, where A is an organic cation, M is a transition metal and X is a halide (Cl⁻, Br⁻ or I⁻) [13–22]. But here is the exciting part: adding organic ligands takes the adaptability and usefulness of materials to the next level. When you combine bromide and chloride halides in organic–inorganic hybrid materials, you open the door to create a wide range of structures, from zero-dimensional [23], one-dimensional [24] and even two-dimensional structures [25]. These hybrid materials, featuring a mix of halides, can crystallize in diverse space groups and assume various crystal systems. Interestingly, they often display occupational disorder, where bromide and chloride ions are present in the same positions, adding complexity to their structure [23,25,26]. Cobalt (Co) has emerged as a pivotal transition metal in the realm of coordination chemistry, owing to its versatile coordination environments and the rich array of properties it imparts to hybrid materials. Recent studies have highlighted the synthesis and characterization of cobalt-based coordination polymers and complexes that exhibit remarkable structural diversity [27,28]. Moreover, pyridine-based macrocyclic ligands have been synthesized to form cobalt(II) complexes, which have revealed interesting structural and magnetic characteristics [27,29,30]. Furthermore, the exploration of cobalt(II) coordination polymers has led to the discovery of materials exhibiting single-ion magnet (SIM) behaviour, which is of considerable interest for applications in quantum computing and high-density information storage [31]. Specifically, two hybrid materials have been studied, both containing cobalt (II) as the transition metal, 4-dimethylaminopyridine as a templating agent and either chloride or bromide as the halide [32,33]. These compounds (C_7_H_11_N_2_)_2_[CoBr_4_] and (C_7_H_11_N_2_)_2_[CoCl_4_] are supramolecular or one-dimensional and crystallize in the *C*2/*c* and P−1 space groups. What makes these mixed halide compounds truly special is their unique combination of optical, magnetic and biological properties, which set them apart from other materials. This study takes a closer look at a brand-new mixed halide hybrid material, exploring its chemical synthesis, thermal behaviour, crystal structure and its optical and biological activities. This compound (1) brings together cobalt as the transition metal, 4-(dimethylamine)pyridine (DMAP) as the organic framework and bromide and chloride as the halide components.

## 2. Theoretical part

### 2.1. X-ray data collection

The selection of an appropriate single crystal of the title compound was completed under a polarizing microscope and mounted on a cactus needle. The crystal was then placed on a D8 VENTURE Bruker AXS area-detector diffractometer, equipped with graphite monochromatic Mo Kα radiation (λ = 0.71073 Å) and cooled to 150(2) K. The space group was determined using the automated search function in WinGX [34]. The resolution of the structure was developed in the centrosymmetric space group *C*2/*c* using SHELXT 2018 [35]. The structure was further refined using SHELXL-2019 [36]. Hydrogen atoms of the organic cation were positioned geometrically and constrained to ride on their parent atoms, with idealized C–H and N–H bond lengths, respectively, of 0.95 and 0.84 Å. Additional details on data collection and refinement procedures are summarized in electronic supplementary material, table S1. The final atomic coordinates were used to derive bond distances and angles, and potential hydrogen bonds presented respectively in electronic supplementary material, tables S2, S3 and S4. The figures were created using Diamond 3 software [37].

### 2.2. Computational details

Density functional theory (DFT) was used using Gaussian 09 software [38], in order to optimize the molecular geometry and calculate vibrational wavenumbers of (1). Specifically, we utilized the B3LYP functional, which combines Becke’s three-parameter hybrid exchange functional (B3) [39] with the Lee–Yang–Parr (LYP) correlation functional [40]. The optimization process was unconstrained, leveraging the analytical gradient procedure in Gaussian 09. All structural parameters were freely adjusted, yielding an optimized geometry upon convergence. The absence of imaginary frequencies in the vibrational wavenumber calculations confirmed that the optimized structure corresponds to a true energy minimum.

## 3. Biological study

### 3.1. DPPH radical scavenging assay

To evaluate the sample’s capability in scavenging 1,1-diphenyl-2-picrylhydrazyl (DPPH) radicals, a previously reported method was employed [41]. Various sample concentrations were prepared ranging from 62.5 to 1000 μg ml^−1^. In order to assess the antioxidant capacity of the synthesized compound (1), 1.5 ml of DPPH solution (0.004%) was added to a test tube containing a volume of 0.5 ml of the sample. Different concentrations were tested. Before measuring its absorbance at 517 nm, using a UV spectrophotometer, the obtained mixture was left in the dark for 30 min.

The experiment was performed in triplicate, and the percentage of inhibition (%) was calculated using the following equation:


(3.1) $$Inhibition\;\left(\%\right)=\frac{A_{1}-A_{0}}{A_{1}}\times 100,$$

where A_1_ is the absorbance of the control and A_0_ is the absorbance of each sample. For each sample, the free radical scavenging activity was evaluated by comparison with ascorbic acid as the reference standard compound.

### 3.2. Ferric reducing antioxidant power

The ferric reducing antioxidant power (FRAP) assay is a widely accepted method for evaluating the antioxidant capacity of a compound in qualifying oxidative damage generated by reactive oxygen species (ROS). To assess the reducing power of bis(4-dimethylaminopyridinium)bromo-chlorocobaltate(II) (1), we employed the process developed by Oyaizu [42] with slight modifications. We prepared various concentrations of the synthesized complex and ascorbic acid, ranging from 0.2 to 1 mg ml^−1^. Each sample was then mixed with 2.5 ml of phosphate buffer (0.2 M, pH 6.6) and 2.5 ml of 1% potassium ferricyanide (K_3_Fe(CN)_6_). The resulted mixture has been subjected to incubation at a temperature of 50°C for 20 min, followed by cooling. Next, 2.5 ml of 10% trichloroacetic acid (TCA) was added, and the mixture was centrifuged at a speed of 3000 r.p.m. for 10 min.

The resulting supernatant (2.5 ml) was transferred to a tube containing 0.5 ml of ferric chloride (FeCl_3_), and the absorbance was measured at 700 nm. All tests were performed in triplicate. A higher absorbance value reveals an important reducing power of the sample, suggesting its potential antioxidant activity [43].

### 3.3. Anti-bacterial activity

The anti-bacterial activity of the synthesized compound was tested *in vitro* against *Salmonella typhi, Bacillus subtilis, Pseudomonas aeruginosa, Escherichia coli* and *Staphylococcus aureus* bacterial strains using the agar well diffusion method [44,45]. All the above bacteria were grown in Mueller−Hinton broth for 18−24 h at 37°C. For the experiment, 10 μl of the synthesized compound (0.5 mg ml^−1^) was used to impregnate sterile 6 mm discs, which were then placed onto agar plates containing the bacterial test strains. Ampicillin, a standard anti-bacterial drug, was used as a positive control. The inoculated Petri dishes were incubated overnight at a temperature of approximately 37°C, and anti-bacterial activity was evaluated by measurement of the diameter of the inhibition zone (mm) using a flat 1 cm ruler. A comparison between the growth inhibition results and those of the reference drug was developed.

### 3.4. Anti-diabetic activity: α-amylase inhibition

We performed the α-amylase inhibition assay in a 96-well plate, following the protocol described by Bernfeld [46]. Acarbose was used as the standard inhibitor for comparison. Different concentrations of the tested compound (0, 25, 50 and 100 μg ml^−1^) were prepared in advance. In brief, the α-amylase inhibition assay involved mixing 20 μl of α-amylase, 10 μl of 2 mM phosphate buffer (pH 6.9) and 10 μl of the sample solution. The mixture was pre-incubated at 35°C for 20 min, followed by the addition of 10 μl of 1% starch solution. After incubation for 5 min at a temperature of approximately 95°C, the reaction was stopped by adding 50 μl of dinitrosalicylic acid (DNS) reagent. A microplate reader was then used to measure the absorbance at 540 nm. All tests were performed in triplicate. The calculation of the percentage inhibition of the α-amylase enzyme was established using the equation


(3.2) $$Inhibition\;\left(\%\right)=\frac{Control-Test}{Control}\times 100.$$

### 3.5. Anti-inflammatory activity

Protein denaturation occurs when proteins lose their native conformation due to various external stressors, such as extreme pH, high salt concentrations, organic solvents, or elevated temperatures. A compound’s ability to inhibit protein denaturation is indicative of its potential anti-inflammatory properties. We assessed this activity *in vitro* using the egg albumin denaturation assay, adapting a previously described method [47]. The reaction mixture (5 ml) comprised 0.2 ml of egg albumin, 2.8 ml of phosphate-buffered saline (PBS, pH 6.4) and varying concentrations of the test compound (0, 20, 40, 60 and 100 μg ml^−1^). Double-distilled water served as a control. After incubation at 37 ± 2°C for 15 min, the mixtures were heated to 70°C for 5 min and then cooled. Absorbance measurements were taken at 660 nm. Diclofenac was used as the reference standard.

## 4. Results and discussions

### 4.1. Structure description

**Figure 1.**
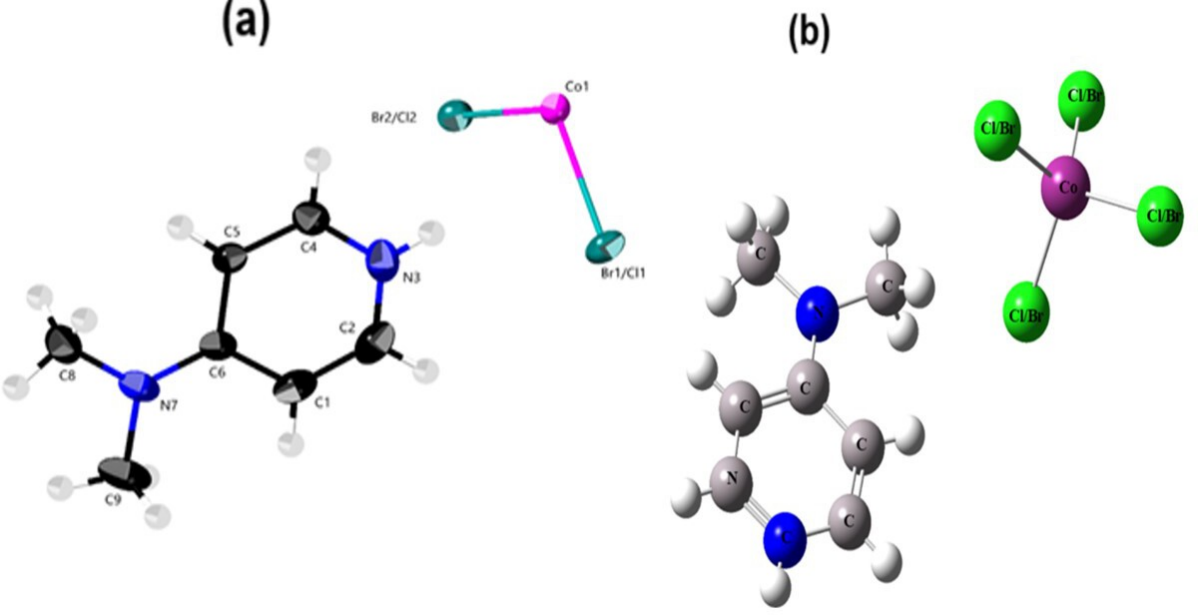
(a) The asymmetric unit of (1), (b) B3LYP/LanL2DZ optimized geometry of (1).

The investigated cobalt complex, bis(4-dimethylaminopyridinium)bromo-chlorocobaltate(II) (C_7_H_11_N_2_)_2_[CoBr_2.28_Cl_1.72_], crystallizes in the centrosymmetric *C*2/*c* space group of the monoclinic system, with four formula units per unit cell. The unit cell parameters are as follows: a = 10.3726(6) Å, b = 11.9706(7) Å, c = 16.8926(9) Å, β = 104.814(2)° and V = 2027.8(2) Å³ (electronic supplementary material, table S1). The incorporation of various DFT methodologies, particularly the B3LYP/LanL2DZ level of theory, plays a crucial role in deepening the understanding of the structural, electronic and bonding characteristics of the investigated cobalt-based hybrid halide compound, (C_7_H_11_N_2_)_2_[CoBr_2.28_Cl_1.72_]. As illustrated in figure 1b, the DFT-optimized geometry closely aligns with the experimental crystallographic data (figure 1a), thus validating the theoretical approach and reinforcing its predictive power. Through such computational modelling, it is possible to gain insights into the local coordination environment of the Co(II) centre and the influence of mixed halide occupancy (Br⁻/Cl⁻) on the overall stability and electronic distribution within the [Co(Br/Cl)₄]²⁻ cluster.

**Figure 2.**
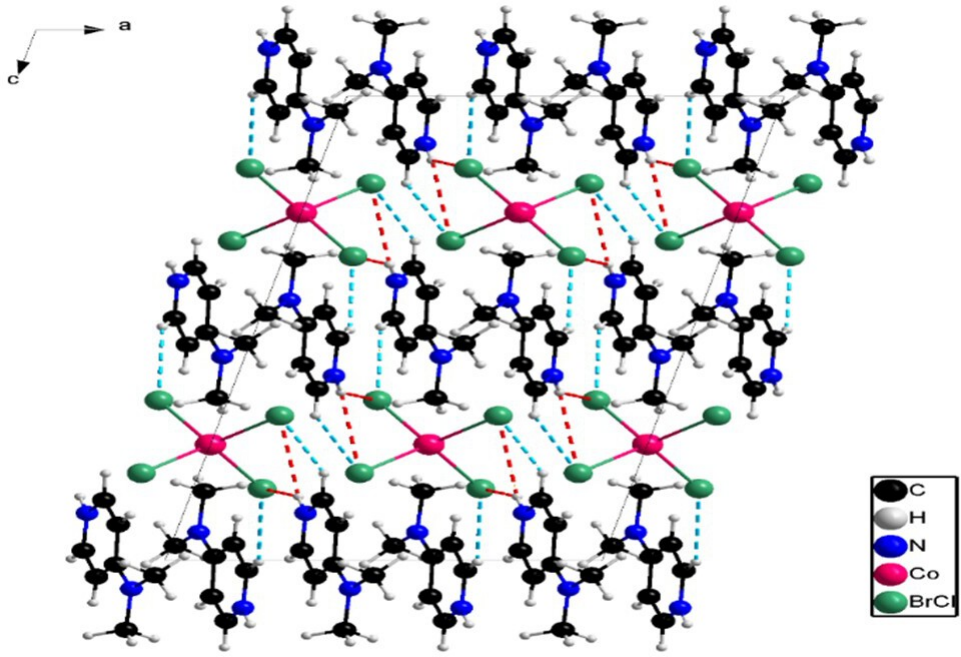
Projection of the structure along the crystallographic b axis and the hydrogen bonding between the different entities: N–H…Br/Cl bonds (in red); C–H…Br/Cl bonds (in blue).

Furthermore, DFT calculations help elucidate the nature of non-covalent interactions, such as hydrogen bonding (N–H…Br/Cl and C–H…Br/Cl), which govern crystal packing and contribute to the supramolecular architecture (figure 2). These interactions are often difficult to characterize in detail through experimental means alone. In systems exhibiting occupational disorder like the partial substitution of bromide and chloride in equivalent sites, DFT proves indispensable in assessing the energetic preference of various configurations and in modelling the subtle structural perturbations caused by halide mixing. As supported by prior studies on mixed halide hybrid systems [23,26,48], theoretical tools are particularly valuable for interpreting disorder phenomena and their effect on material properties. Notably, all atoms, except for the cobalt atom, occupy general positions (Wyckoff site: 4e). The formula unit comprises inorganic [Co(Br/Cl)₄]²⁻ tetrahedral anions and two protonated organic amines (C_7_H_11_N_2_)^+^. These components are linked through hydrogen bonding interactions, specifically N–H…Br/Cl and C–H…Br/Cl, as depicted in figure 2. Analogous to mixed halide hybrid materials [23,26,49], this structure displays occupational disorder. Bromide and chloride, specifically, share the same equivalent positions, with refined occupancy levels of 0.558 and 0.582 for bromide and 0.442 and 0.418 for chloride at the first and second sites, respectively (electronic supplementary material, table S2), while the remaining sites are determined by symmetry. As shown in figure 2, the Co(II) ion is coordinated by four Br/Cl halide ligands, forming a distinct coordination environment. To elucidate the exact geometry of the cobalt polyhedron, we computed the τ₄ parameter, a metric that differentiates between square planar (τ₄ = 0) and tetrahedral (τ₄ = 1) coordination geometries. This calculation was performed using a previously established formula [50]


(4.1) $$\tau_{4}\;=\frac{360-\;\alpha +\beta }{360-2\theta }.$$

The τ₄ parameter calculation involves the two largest bond angles (α and β) within the polyhedron, as well as the ideal tetrahedral bond angle (θ = 109.5°). For the compound under investigation, α = 113.74° and β = 113.309°, yielding a τ₄ value of 0.943. This result indicates that the Co(II) polyhedron exhibits a tetrahedral geometry. Within the [Co(Br/Cl)_4_]²⁻ tetrahedra, the Co–Br/Cl bond lengths range from 2.3589(3) to 2.3644(3) Å, while the Br/Cl–Co–Br/Cl bond angles span 103.677(10)°–113.74(2)° (electronic supplementary material, table S2). These findings suggest a slight distortion of the tetrahedron. Notably, the [Co(Br/Cl)_4_]²⁻ tetrahedra remains isolated, with the shortest Co–Co distance measuring 7.9197(5) Å. It should be noted that the experimental X-ray diffraction (XRD) powder pattern for the compound matched well with the simulated one from single crystal structure data indicating that the compound was isolated as a single phase, as shown in electronic supplementary material, figure S4.

**Figure 3.**
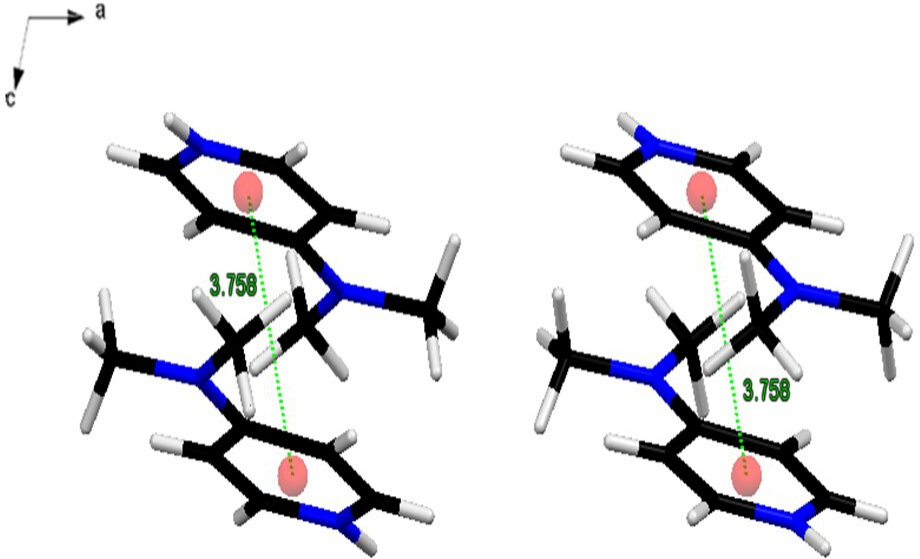
Offset-face-to-face interactions motifs (π–π stacking, broken lines) in the cation chains.

The organic cations are integrated into the inorganic framework to counterbalance the negative charge of the anionic component. In this structure, the organic layers consist of protonated DMAP cations, which are interconnected through aromatic–aromatic (π–π) interactions. These π–π interactions occur in a parallel-displaced arrangement, with a measured interplanar distance of 3.758 Å between the two pyridine rings, as illustrated in figure 3.

Each organic cation participates in N–H…Br/Cl and C–H…Br/Cl hydrogen bonding, which plays a primordial role in preserving the structural cohesion. These interactions arise from intermolecular hydrogen bonds between the organic and inorganic layers, contributing to the stability of the overall structure (figure 2). For the N–H…Br/Cl hydrogen bonds, the N…Br/Cl distances range from 3.4075(18) to 3.511(2) Å, while the N–H…Br/Cl bond angles vary between 133° and 142°. In the C–H…Br/Cl interactions, the C…Br/Cl distances fall within 3.555(2) to 3.8169(19) Å, with bond angles ranging from 124° to 165° (electronic supplementary material, table S4).

The crystal structure of the title compound (1) which crystallizes in the monoclinic *C*2/*c* space group, reveals notable similarities with structurally related halometallate complexes such as bis[4-(dimethylamino)pyridinium]tetrabromidocobaltate(II) [13], which also adopts the same *C*2/*c* space group. These materials are part of a broader family of [CoX_4_]²⁻-based hybrids, including chloridocuprates (bis[4-(dimethylamino)pyridinium]tetrachloridocuprate) [48], chloridomanganates (bis[4-(dimethylamino)pyridinium]tetrachloridomanganate) [51] and chloridoferrates (4-(Dimethylamino)pyridinium tetrachloridoferrate [52], where X = Cl⁻ or Br⁻, and all share a common feature: a tetrahedral coordination geometry around the central metal ion, stabilized by a supramolecular network of hydrogen bonding and π–π interactions. In all cases, the tetrahedral geometry of the anionic units is preserved, as supported by τ₄ values close to 1, such as the value of 0.943 calculated for the present Co(II) complex, indicating a slightly distorted tetrahedral arrangement. The structural framework is further reinforced by extensive N–H···Br/Cl and C–H···Br/Cl hydrogen bonds, involving both halide ligands and the protonated DMAP cations, which are positioned on general Wyckoff sites (4e) in all referenced structures. In addition, the π–π stacking interactions between adjacent DMAP cations, observed as offset face-to-face motifs with interplanar distances around 3.76 Å, closely resemble the supramolecular arrangements found in related [CuCl_4_]^2^⁻ and [MnCl_4_]^2^⁻ systems. In contrast, the structurally related compound (C_7_H_11_N_2_)_2_[CoCl_4_] [33] crystallizes in the triclinic system (space group *P*−1), reflecting a lower symmetry arrangement. Despite maintaining the same tetrahedral coordination environment around the Co(II) centre and comparable hydrogen bonding patterns involving N–H···Cl and C–H···Cl interactions, the reduced symmetry in the triclinic system results in more pronounced variations in bond angles and spatial orientations of the organic cations. This comparison highlights how the symmetry of the crystal system monoclinic *C*2/*c* versus triclinic *P*−1 influences the degree of structural order, packing efficiency and overall molecular arrangement.

### 4.2. Hirshfeld surface analysis

**Figure 4.**
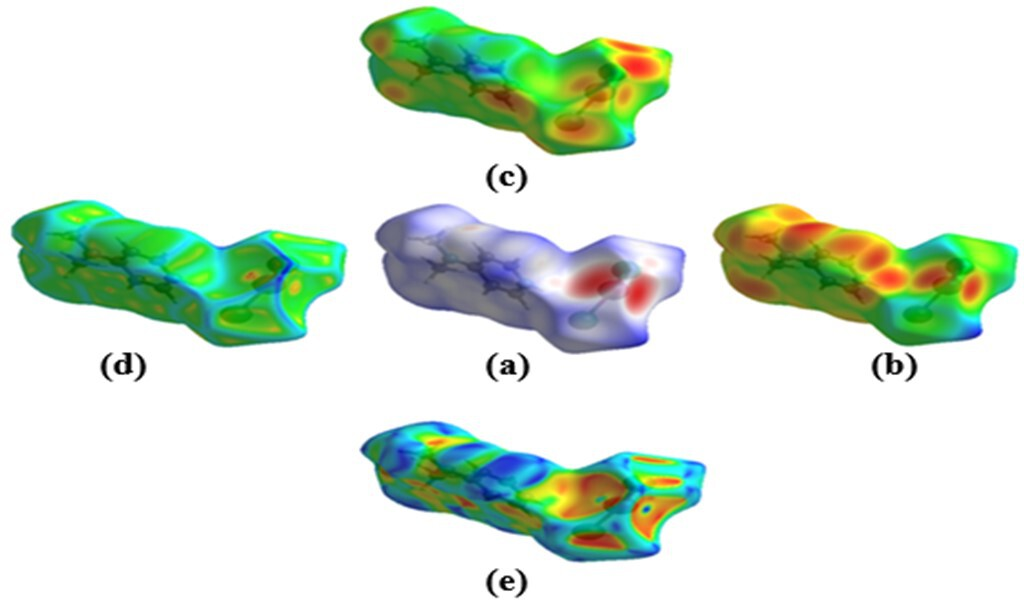
Hirshfeld surface analysis of (1): (a) d_norm_ surface, (b) d_i_, (c) d_e_, (d) curvedness and (e) surface index.

To better understand the intermolecular interactions within the crystal structure of (1), Hirshfeld surface analysis was performed using d_norm_, curvedness and shape index mappings. These allow for a deeper visualization of contact distribution and topological variation across the molecular surface. The d_norm_ surface (figure 4a) clearly highlights red spots indicating short interatomic contacts, mainly attributed to N–H…Br/Cl and C–H…Br/Cl hydrogen bonds, which are the dominant stabilizing interactions in the crystal packing. The curvedness map (figure 4d) reveals wide flat regions on the molecular surface, reflecting π–π stacking zones between pyridinium rings, consistent with structural observations (figure 3).

**Figure 5.**
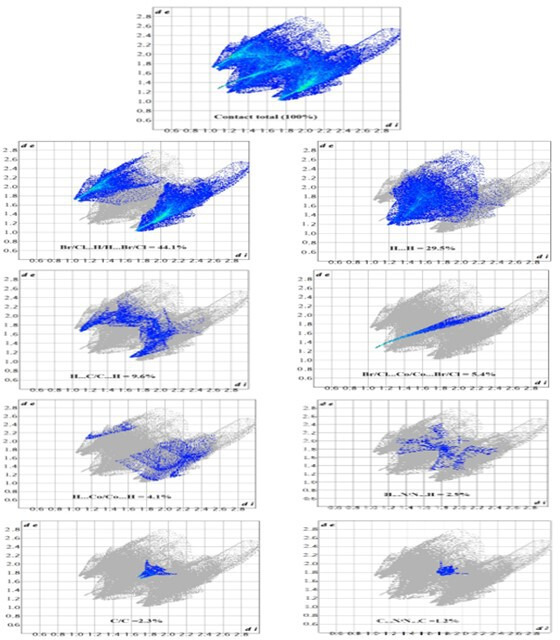
The two-dimensional-fingerprint plots for (1).

Furthermore, two-dimensional fingerprint plots (figure 5) quantify the relative contributions of various contacts. The Br/Cl…H/H…Br/Cl interactions account for 44.1% of the surface contacts, followed by H…H interactions (29.5%), indicating a high degree of hydrogen bonding. Other minor interactions include H⋯C/C⋯H (9.6%), H⋯Co/Co⋯H (4.1%) and H⋯N/N⋯H (2.9%).

To provide context, we compared this data with two analogous compounds: the chloride-only (C_7_H_11_N_2_)_2_[CoCl_4_] [33] and the bromide-only (C_7_H_11_N_2_)_2_[CoBr_4_] [13] (electronic supplementary material, table S5). The mixed halide system exhibits intermediate interaction percentages, combining features from both ends of the halide spectrum.

As shown, the mixed halide compound maintains a balanced profile between bromide-rich and chloride-rich analogues, which probably contributes to its optimized crystal packing and stability. It should be noted that the values reported for (C_7_H_11_N_2_)_2_[CoBr_4_] are approximate, as no detailed Hirshfeld surface analysis data could be found in the literature for this compound. These figures are likely estimations based on structural trends observed in similar systems.

### 4.3. Vibrational study

**Figure 6.**
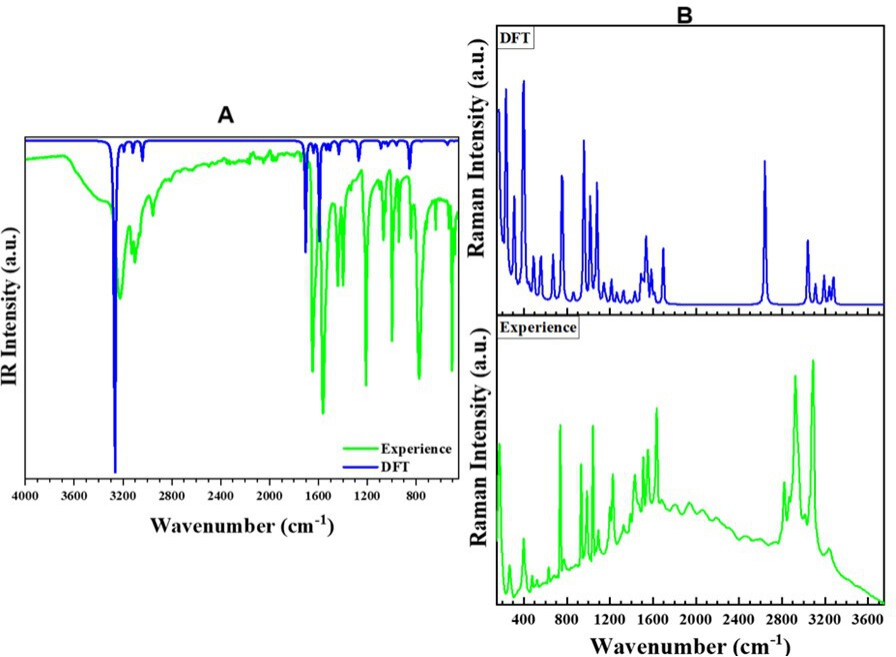
(A) Superposition of the experimental (blue) and DFT-computed (green) IR spectra of (1) in the region 400−4000 cm^-1^, (B) Superposition of the experimental (blue) and DFT-computed (green) Raman spectra of (1) in the region 50−3500 cm^-1^.

Figure 6, including both experimental and calculated data, illustrates the infrared and Raman spectra. The detailed assignments of the observed vibrational bands in these spectra are provided in electronic supplementary material, table S6. A comparison between figure 6A,B reveals a strong correlation between the experimental and simulated spectra, confirming the reliability of the theoretical calculations in reproducing the spectral features. The vibration modes associated with the organic cation are clearly observed in both spectra and are thoroughly described in electronic supplementary material, table S6. The band assignments were informed by a review of prior theoretical and experimental investigations described in previous works for analogous compounds [53–56].

The infrared spectrum displays prominent bands at 3231, 3128, 3102 and 2960 cm^−1^, attributed to asymmetric and symmetric stretching vibrations of N–H and C–H bonds. The absorption band at 1648 cm^−1^ is indicative of N–H bending. The pyridine ring’s stretching modes, corresponding to C = C and C = N bonds, are observed at 1562 and 1443 cm^−1^. Additionally, the band at 1211 cm^−1^ is assigned to ν (C–N) and ν (C–C) vibrational modes. The absorption band at 1069 cm^−1^ is associated with δ (C–C) bending. The remaining bands between 1000 and 500 cm^−1^ are attributed to out-of-plane bending modes of γ (C–C), γ (C–H) and γ (C–N) bonds. In the Raman spectrum, Co-Br/Cl stretching and deformation modes are observed at 386, 271, 204 and 164 cm^−1^, corresponding to asymmetric and symmetric vibrations.

## 5. Thermal behaviour

**Figure 7.**
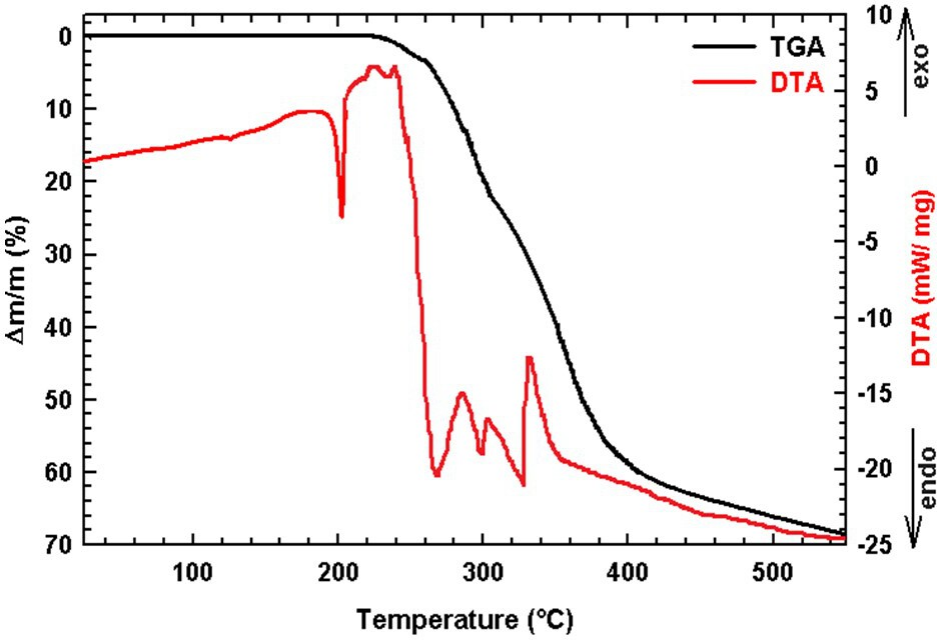
Simultaneous thermogravimetric and differential thermal analysis of (1).

**Figure 8.**
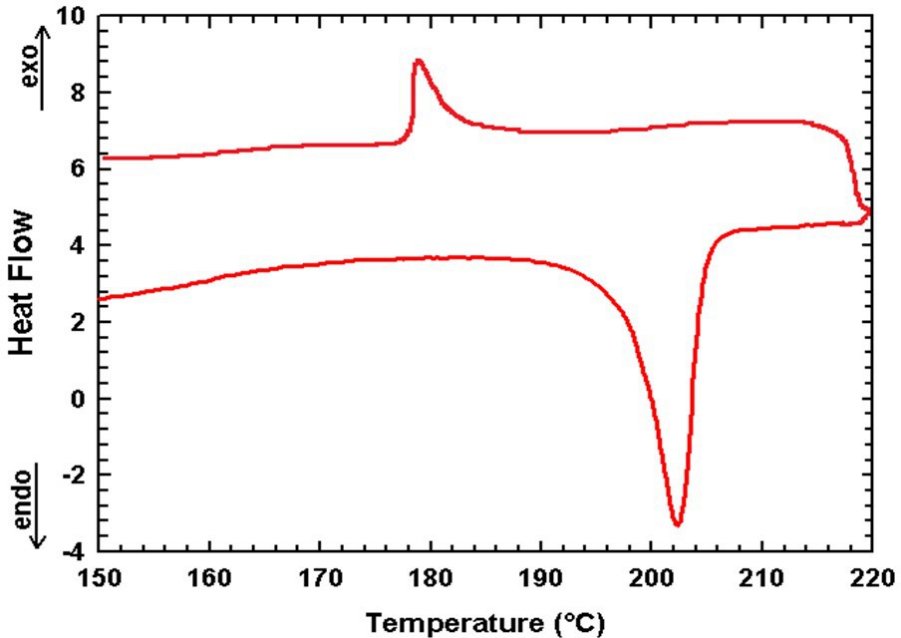
Differential scanning calorimetry measurements of (1).

The thermal behaviour of the mixed halide compound bis(4-dimethylaminopyridinium)bromo-chlorocobaltate(II) (1) (C_7_H_11_N_2_)_2_[CoBr_2.28_Cl_1.72_] was comprehensively examined using thermogravimetric analysis (TGA), differential thermal analysis (DTA) (figure 7) and differential scanning calorimetry (DSC) (figure 8). The thermal decomposition of the compound (1) occurs through four major steps, each associated with specific temperature ranges and corresponding mass losses. In the first step (223−310°C), the loss of one organic moiety (C_7_H_10_N_2_) occurs, with a theoretical mass loss of 22.11%, while the experimentally observed loss is 22.05%, indicating good agreement. This phenomenon is accompanied by an endothermic peak observed on the DTA curve at 230°C. The second step between 305°C and 361°C corresponds to the release of the second organic unit (C_7_H_10_N_2_), with a theoretical mass loss of 20.62%, and an experimental value of 20.40%. This decomposition process is accompanied by endothermic peaks observed at 313°C and 350°C. In the third stage, heating between 361°C and 405°C results in the elimination of hydrogen halides (HBr and HCl), with a calculated mass loss of 16.23%, compared with an experimental value of 15.98%. Finally, in the temperature range of 410°C−550°C, halogen molecules Br_2_ and Cl_2_ are released, leaving behind cobalt(II) oxide (CoO) as the final residue. Based on the thermal analysis data, the thermal decomposition of the compound may be expressed as follows:


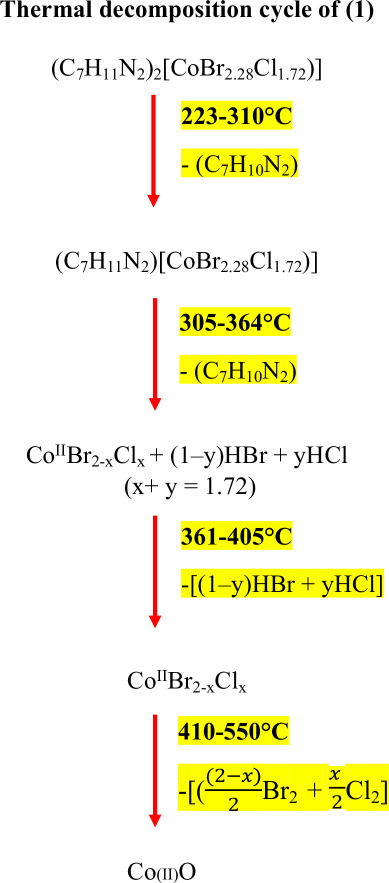


### 5.1. Optical properties

**Figure 9.**
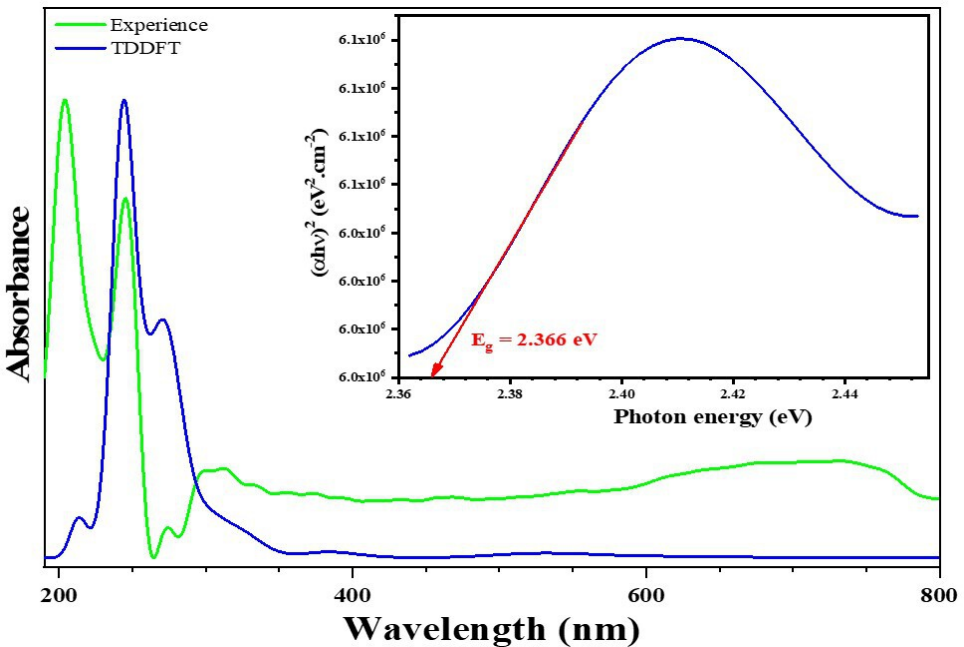
The energy gap and optical absorption spectra of (1) at room temperature.

Figure 9 corresponds to the experimental and calculated absorption spectra of the (C_7_H_11_N_2_)_2_[CoBr_2.28_Cl_1.72_] complex, including four different absorption bands. The three prominent bands in the UV region, centred at 205, 246 and 298 nm, are attributed to a combination of π→π* and n→π* transitions within the DMAP organic cation, as well as ligand-to-metal charge transfer (LMCT) from the Br/Cl orbital to the Co(II) 3d orbital, consistent with prior research [57,58]. Time-dependent DFT (TD-DFT) calculations predict three absorption peaks at 213, 244 and 268 nm, which are coherent with the experimental bands, as illustrated in figure 9). Furthermore, a weaker band at 701 nm corresponds to a d–d electronic transition band, similar to those observed in other cobalt-based hybrid materials [59–61]. The optical properties of this material are crucial for technological applications, particularly the optical absorption coefficient (α) and band gap energy (*E_g_*). The following equation, showing the relationship between the absorption coefficient and incident photon energy (hν), allows the estimation of the band gap [62]:


(5.1) $${\left(\alpha h\nu \right)}^{2}=A\left(h\nu -E_{g}\right).$$

The band gap energy ($E_{g}$) can be extracted from the equation, where *A* is a constant and *hν* represents the incident photon energy.

The band gap value of 2.366 eV is obtained from the intersection of the steeply declining region (region of maximum slope) with the baseline, as depicted in figure 9. This value categorizes the material as a semiconductor. This result is comparable to the published works of similarly complexes in literature [63–66].

### 5.2. Biological activities

#### 5.2.1. DPPH scavenging effect

**Figure 10.**
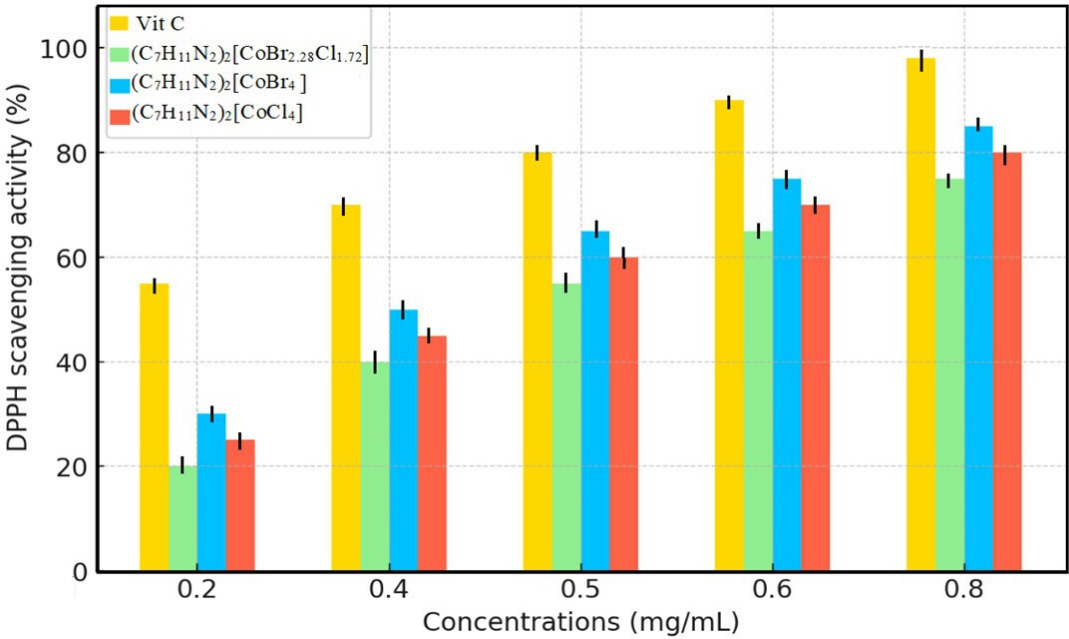
Scavenging activity of (1), (2), (3) and vitamin C against the radical DPPH.

The antioxidant activity of the synthesized complex (C_7_H_11_N_2_)_2_[CoBr_2.28_Cl_1.72_] (1) was evaluated in relation to its hydrogen-donating or radical-scavenging ability using the stable free radical DPPH. In this method, antioxidant substances reduce DPPH radicals (violet) to yellow-coloured diphenylpyrrilhydrazine. Based on the results in figure 10, the synthesized compound displayed a high DPPH radical scavenger potential (IC₅₀ = 0.53 ± 0.015 mg ml^−1^) compared with the positive reference, ascorbic acid (IC₅₀ = 0.142 ± 0.004 mg ml^−1^). The antioxidant activity of the tested compound and standard was observed to rise in proportion to the dose. As illustrated in the bar chart, the scavenging activity of (C_7_H_11_N_2_)_2_[CoBr_4_] (2) and (C_7_H_11_N_2_)_2_[CoCl_4_] (3) increased progressively with concentration, demonstrating significant radical scavenging potential. However, ascorbic acid (vitamin C) exhibited the highest activity across all concentrations, confirming its superior antioxidant efficacy. The mixed halide complex (1) showed competitive antioxidant activity, though slightly lower than ascorbic acid. The presence of cobalt and halide ions appears to enhance the radical-scavenging ability, possibly due to their redox properties and capacity to stabilize free radicals.

The observed scavenging potential can be ascribed to the proton diffusion capacity and the unique composition of (1), which harbours reactive sites comprising carbon and nitrogen atoms. These sites can facilitate proton donation or electron acceptance, thereby enhancing the compound’s scavenging ability. Notably, analogous compounds incorporating cobalt and/or bromine have exhibited similar properties [67,68]. Substances exhibiting such activity can be classified as antioxidants and function as free radical scavengers. Moreover, cobalt plays a key role in the antioxidant activity of vitamin B₁₂ by participating in redox reactions and catalysing biochemical processes. Given these findings, the studied cobalt-based complex presents a promising avenue for potential therapeutic applications in managing oxidative stress-related degenerative disorders.

#### 5.2.2. Ferric-reducing antioxidant assay

**Figure 11.**
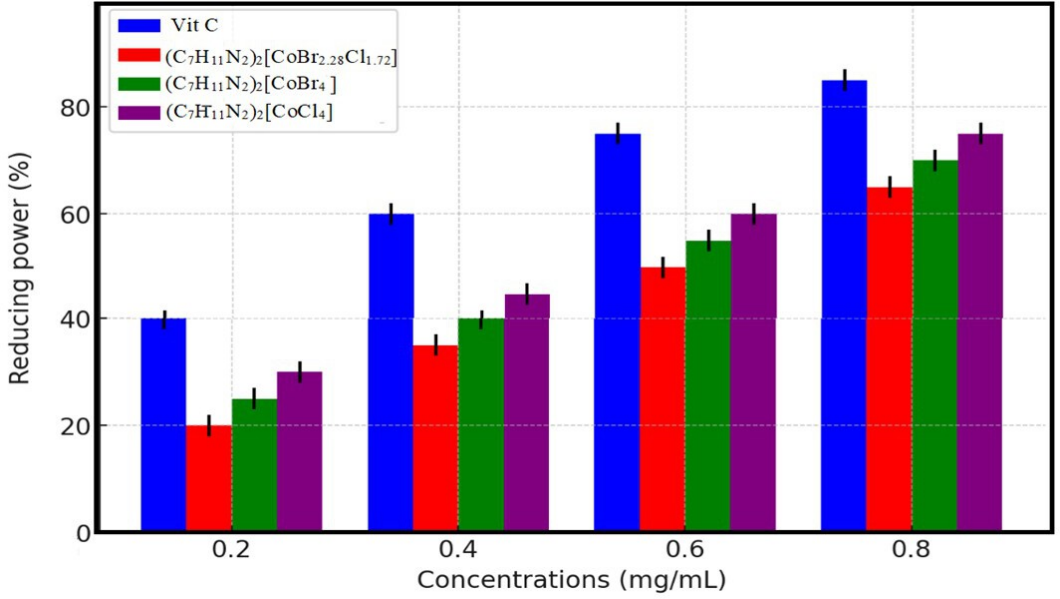
Ferric reducing antioxidant power assay of vitamin C, (1), (2) and (3).

The antioxidant activity of the synthesized complex (1) was assessed based on its ability to reduce Fe³^+^ to Fe²^+^, a key indicator of electron transfer capacity and antioxidant potential [69]. Figure 11 illustrates the FRAP of the synthesized compound, compared with ascorbic acid, a reference antioxidant. The data reveal a dose-dependent escalation in reducing activity, where elevated concentrations of the compound correlate with enhanced reduction capacity, demonstrating its potent antioxidant properties.

Vitamin C exhibited the highest reducing power across all tested concentrations, confirming its strong antioxidant properties (IC50 = 0.314 ± 0.01 mg ml^−1^). Among the synthesized compounds, (C_7_H_11_N_2_)_2_[CoBr_2.28_Cl_1.72_] displayed notable reducing ability, with an IC50 value of 0.71 ± 0.021 mg ml^−1^. The reducing activity of (C_7_H_11_N_2_)_2_[CoBr_4_] (2) and (C_7_H_11_N_2_)_2_[CoCl_4_] (3) followed a similar trend but was slightly lower, suggesting that the presence of mixed halides (Br and Cl) enhances electron donation.

The pyridine ring’s electron delocalization probably plays a key role in this behaviour. By distributing electrons throughout the ring, the electron density increases, making it easier to reduce ferric ions to their ferrous state [70]. These findings are consistent with previous reports on the antioxidant properties of metal-based complexes [67,71], reinforcing the potential of cobalt coordination compounds as effective reducing agents. The presence of Co²^+^ in the complex probably contributes to its redox activity, supporting its potential role in oxidative stress regulation and biomedical applications.

#### 5.2.3. Anti-bacterial activity and structure–activity relationship

**Figure 12.**
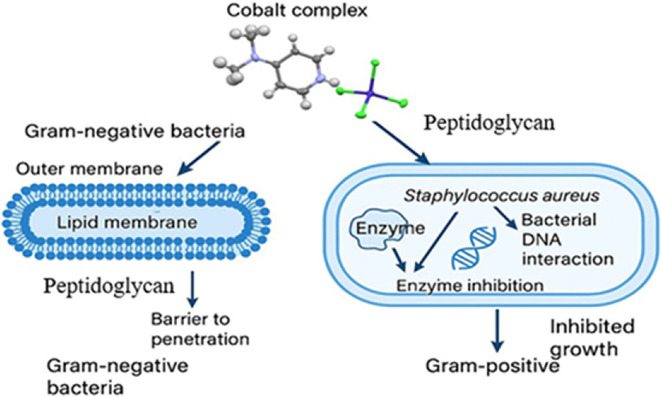
Pathway of anti-bacterial action of cobalt complexes.

**Figure 13.**
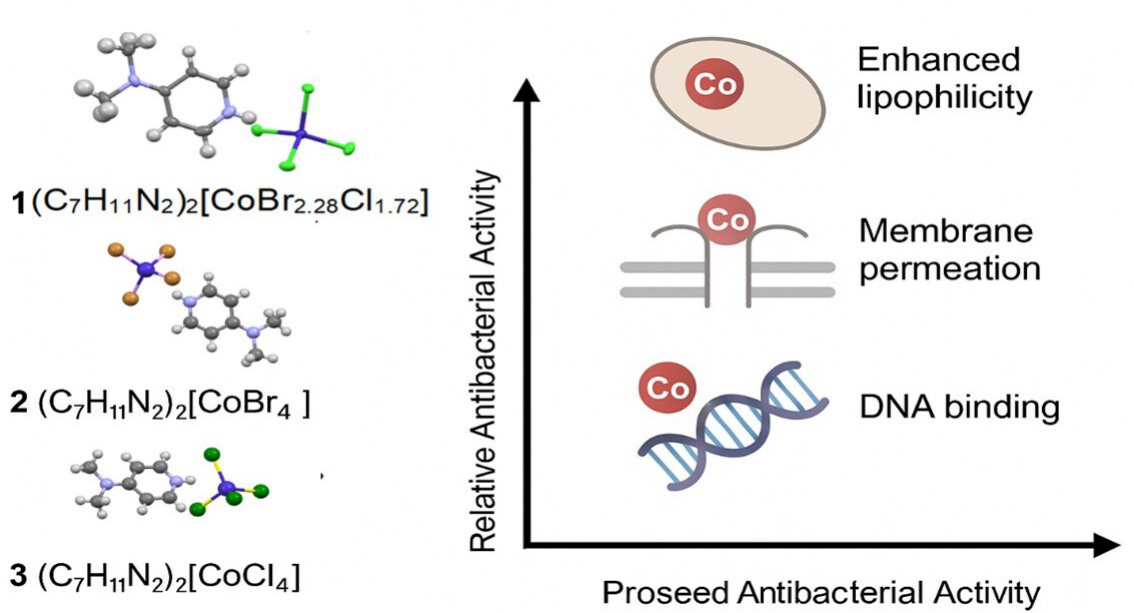
Structure–activity relationship (SAR) of cobalt complexes (1), (2) and (3).

The minimum inhibitory concentration (MIC) and zone of inhibition (ZOI) of the synthesized cobalt-based salts (1), (2) and (3) were investigated against both Gram-positive and Gram-negative bacterial strains, including *S. aureus*, *B. subtilis*, *E. coli*, *Klebsiella pneumoniae*, *S. typhi* and *P. aeruginosa* (figure 12). The antimicrobial performance was compared with the commercial drug Ampicillin under identical conditions. The MIC values (electronic supplementary material, table S7) were determined using the broth microdilution method in accordance with Clinical & Laboratory Standards Institute (CLSI) guidelines. MIC and ZOI analysis revealed that complex (1) displayed superior anti-bacterial activity, particularly against Gram-positive strains, with inhibition zones exceeding 20 mm and MIC values in the range of 62.5−125 µg ml^−1^. This enhanced activity correlates with the mixed halide coordination sphere and electronic environment around the cobalt centre, which probably improves cellular uptake and interaction with biomolecular targets. In contrast, complexes (2) and (3) exhibited moderate to slight activity, especially against Gram-negative bacteria, indicating that halide composition plays a critical role in modulating bioactivity. A comparative (structure–activity relationship; SAR) evaluation (figure 13) suggests that the biological efficacy of these complexes correlates with the halide ligand identity and the electronic environment surrounding the cobalt ion. The presence of both Br and Cl in complex (1) may optimize lipophilicity and redox potential, enhancing membrane permeability and intracellular reactivity. Notably, complex (1) exhibited a MIC value of 62.5 µg ml^−1^ against *B. subtilis* and *S. aureus*, while its effect on *S. typhi* and *P. aeruginosa* was minimal (MIC > 250 µg ml^−1^), probably due to the protective outer membrane in Gram-negative bacteria, which limits penetration by polar or charged compounds. The lipophilic nature of the Co(II) core facilitates interaction with bacterial cell membranes, leading to membrane disruption or enzyme inhibition through ligand exchange or coordination with vital biomolecules such as thiol groups in proteins. Such modes of action are in agreement with DFT-based mechanistic insights reported in similar metal–organic systems [72]. In addition, the planar aromatic 4-dimethylaminopyridinium moiety contributes to π–π stacking interactions with bacterial DNA, potentially inhibiting replication and transcription pathways [73].

#### 5.2.4. α-amylase inhibition

**Figure 14.**
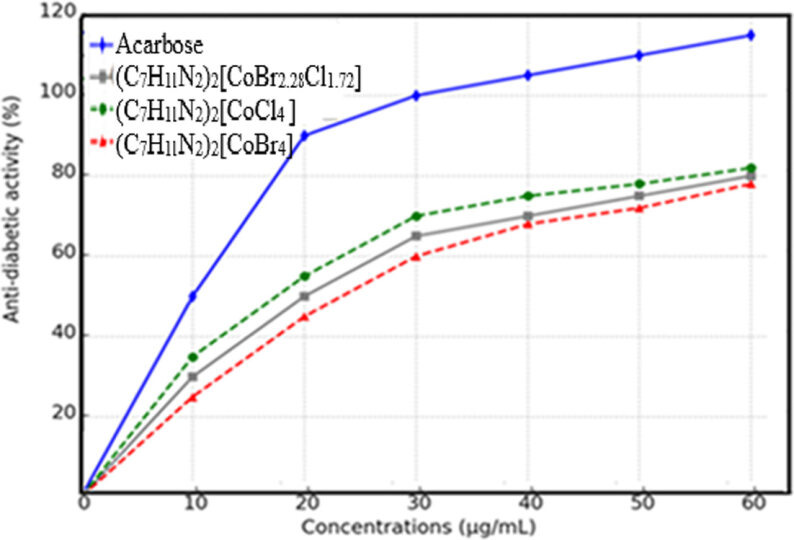
α-amylase inhibition potential of (1), (2) and (3).

Researchers have been exploring the inhibition of α-amylase, a key enzyme responsible for breaking down glycogen and starch, as a potential therapeutic approach for managing carbohydrate-related disorders, particularly diabetes [74]. The anti-diabetic activity of the cobalt complex (C_7_H_11_N_2_)_2_[CoBr_2.28_Cl_1.72_] (1) against α-amylase was evaluated *in vitro*. The findings, summarized in figure 14, reveal that this complex exhibited a remarkable 63% inhibition of α-amylase at a concentration of 100 µg ml^−1^, albeit slightly lower than the standard Acarbose, which achieved 96% inhibition at the same concentration. Notably, the synthesized material demonstrated a dose-dependent inhibitory effect on α-amylase. Further analysis yielded IC50 values of 36.33 ± 2.404 and 17.33 ± 1.453 µg ml−1 for the synthesized compound and standard, respectively. This study participates in the growing body of research exploring the α-amylase inhibitory potential of cobalt complexes [75,76]. The enhanced activity can be attributed to the enzyme’s deactivation through chelation, a process where the compound binds to the enzyme, rendering it inactive. Furthermore, the metal complexes formed were found to reduce blood glucose levels by facilitating the transport of glucose into peripheral tissue cells, thereby activating glucose uptake [77]. The incorporation of bromine atoms into a molecule enhanced its bioactive potential, making bromination an essential tool for creating new drug candidates [78]. This breakthrough discovery could potentially open doors to creating innovative anti-diabetic treatments, offering new hope for managing this debilitating disease. Additionally, figure 14 compares the anti-diabetic activity of (C_7_H_11_N_2_)_2_[CoBr_2.28_Cl_1.72_] (1), (C_7_H_11_N_2_)_2_[CoBr_4_] (2) and (C_7_H_11_N_2_)_2_[CoCl_4_] (3) with Acarbose. The results indicate that both cobalt complexes exhibit significant α-amylase inhibition in a concentration-dependent manner. The (C_7_H_11_N_2_)_2_[CoCl_4_] complex demonstrated a slightly higher inhibitory effect than (C_7_H_11_N_2_)_2_[CoBr_4_], suggesting that chloride coordination may influence enzyme binding affinity. However, both synthesized compounds displayed lower inhibition than Acarbose but remained effective inhibitors. The variation in activity between the bromide and chloride complexes may be attributed to differences in metal–ligand interactions, affecting the binding efficiency to α-amylase. These discoveries support the hypothesis that coordination chemistry plays a primordial role in designing metal-based anti-diabetic agents.

#### 5.2.5. Anti-inflammatory activity

**Figure 15.**
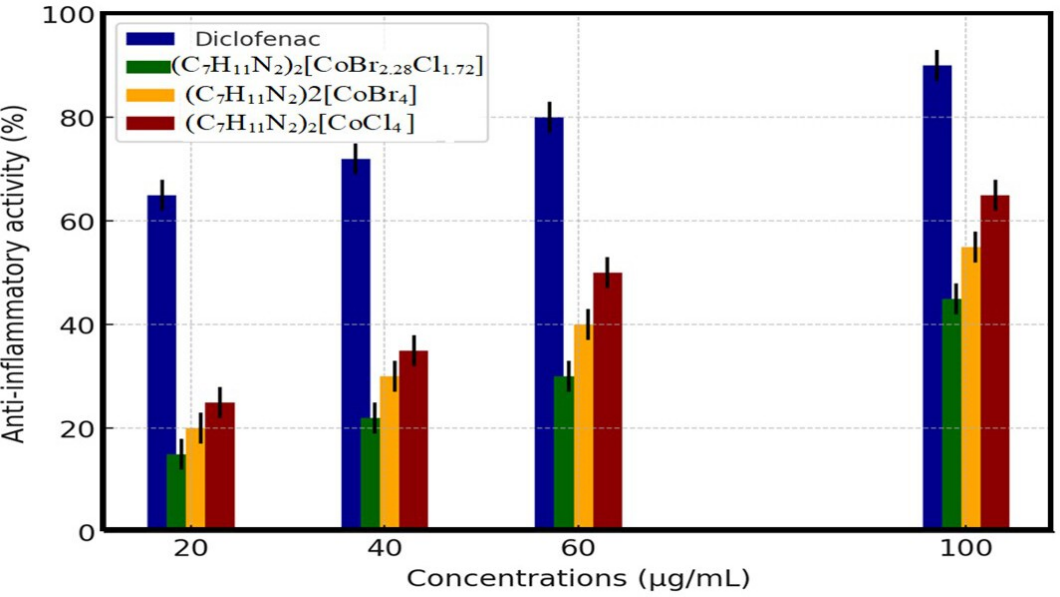
Effect of (1), (2) and (3) on heat-induced protein denaturation.

The anti-inflammatory activity of the prepared compounds was tested in comparison with Diclofenac, a standard non-steroidal anti-inflammatory drug (NSAID). The results, presented in figure 15, demonstrate that (C_7_H_11_N_2_)_2_[CoBr_4_] (2) and (C_7_H_11_N_2_)_2_[CoCl_4_] (3) exhibit moderate anti-inflammatory effects, with inhibition rates increasing proportionally to concentration. Additionally, their activity was compared with Acarbose. At 100 µg ml^−1^, (C_7_H_11_N_2_)_2_[CoCl_4_] (3) displayed the highest inhibition among the synthesized compounds, reaching approximately 65%, while (C_7_H_11_N_2_)_2_[CoBr_4_] (2) showed an inhibition of 55%. Compared with Diclofenac, which exhibited 97% inhibition, the synthesized compounds demonstrated a lower yet significant activity, suggesting their potential as anti-inflammatory agents. The (C_7_H_11_N_2_)_2_[CoBr_2.28_Cl_1.72_] (1) complex showed lower inhibition than both synthesized compounds but still followed the general trend of increasing inhibition with concentration. Protein denaturation is a well-known culprit behind inflammation. To get to the bottom of how these compounds exert their anti-inflammatory effects, the ability to prevent protein denaturation was put to the test. The results were promising: the synthesized compounds showed a notable ability to inhibit heat-induced albumin denaturation, with the highest inhibition rate of 51% achieved at a concentration of 100 µg ml^−1^. Diclofenac, as a reference drug, displayed a maximum inhibition of 97% at the same concentration (figure 15). Protein denaturation involves complex mechanisms, including modifications in electrostatic, hydrogen, hydrophobic and disulfide bonding [79]. Diclofenac, a popular anti-inflammatory medication, works its magic by blocking the activity of the cyclooxygenase enzyme and binding to plasma albumin. This clever binding process prevents or slows down the thermal denzoation of albumin, which is a major contributor to inflammation. In fact, many anti-inflammatory drugs, including diclofenac, rely on this binding mechanism to prevent albumin denaturation and keep inflammation at bay [80]. The compound (C_7_H_11_N_2_)_2_[CoBr_2.28_Cl_1.72_] seems to work in a similar way to NSAIDs, reducing inflammation by suppressing prostaglandin activity. This is achieved by preventing protein denaturation caused by heat, as demonstrated by its ability to inhibit egg albumin denaturation [81]. Interestingly, the higher the percentage inhibition of denaturation, the more potent the anti-inflammatory effect [82]. This suggests that compounds capable of inhibiting protein denaturation may hold significant promise as anti-inflammatory agents.

## 6. Conclusion

This study presents the synthesis, design and comprehensive structural characterization of a novel cobalt(II) complex, (1). The crystal structure reveals a layered architecture composed of inorganic and organic components interconnected through a network of N–H…Br/Cl and C–H…Br/Cl hydrogen bonds. Additionally, π–π stacking interactions contribute significantly to the stability and overall packing of the structure. Thermal analysis identified a single phase transition at 203°C, indicating a distinct thermal behaviour. Infrared and Raman spectroscopy confirmed the protonation of the organic moiety and the coexistence of bromide and chloride ions within the crystal lattice. UV–Vis spectroscopy supported a tetrahedral geometry around the Co²^+^ centre, and the optical band gap was estimated at 2.366  eV (via the Tauc plot), suggesting semiconducting properties. Biological evaluation demonstrated that the cobalt complex exhibits a broad spectrum of bioactivities, including notable antioxidant, anti-bacterial, anti-diabetic and anti-inflammatory effects. These therapeutic properties are attributed to the synergistic coordination of the metal ion with active ligands and the presence of functional sites within the complex. This unique combination of structural and functional features highlights the potential of the synthesized complex as a promising multifunctional agent for addressing oxidative stress, microbial infections, metabolic disorders and inflammation.

## Data Availability

Crystallographic data for (1) have been deposited at the Cambridge Crystallographic Data Centre as supplementary publication CCDC 2415385. Copies of these data can be obtained, free of charge, by application to CCDC, 12 Union Road, Cambridge CB21EZ, UK (fax: +44(0)-1223-336033 or email: deposit@ccdc.cam.ac.uk). Supplementary material is available online [83].
